# Causes of death among young children of female sex workers in three sub-Saharan African countries: a cross-sectional exploratory investigation

**DOI:** 10.3389/fpubh.2025.1696520

**Published:** 2026-02-20

**Authors:** Wendy L. Macias-Konstantopoulos, Revathi Ravi, Brian Willis

**Affiliations:** 1 Center for Social Justice and Health Equity, Department of Emergency Medicine, Massachusetts General Hospital, Harvard Medical School, Boston, MA, United States; 2 Global Health Promise, Portland, OR, United States; 3 Hospital Medicine Unit, Division of General Internal Medicine, Department of Medicine, Massachusetts General Hospital, Harvard Medical School, Boston, MA, United States

**Keywords:** children of female sex workers, hard-to-reach populations, leading causes of child death, low- and middle-income countries, marginalized children, preventable child death

## Abstract

**Introduction:**

Children of female sex workers (CFSW) are a particularly marginalized population of children who experience similar negative health, social, and economic outcomes as their mothers. In low- and middle-income countries (LMIC), the level of vulnerability and adversity is exacerbated by stigma and structural inequities that result in abject poverty, chronic food insecurity, hazardous living conditions, and exposure to violence. The current study aimed to explore the causes of death among CFSW in Kenya, Nigeria, and the Democratic Republic of the Congo (DRC). It seeks to analyze death trends within the context of leading causes of death in the general child population.

**Methods:**

An exploratory, interview-based cross-sectional investigation of the causes of death among CFSW. Interviews with mothers who are female sex workers (MFSW) were conducted across eight cities in the three study countries in 2022 (Kenya, Nigeria) and 2023 (DRC). Data collected included participant sociodemographic information and detailed accounts of the circumstances surrounding the death of their children. Descriptive analysis was employed to organize and present the data.

**Results:**

A total of 188 child deaths reported by 156 MFSW with a mean age of 26.9 years were included for analysis. The overall mean number of deceased children per mother was 1.21, and the highest was in Nigeria (1.32). Newborn and infant deaths (under-1) accounted for 71.3% of all deaths. Neonatal conditions comprised the overall leading cause of death (*n* = 50, 26.6%), followed by infectious diseases (*n* = 41, 21.8%) and malnutrition (*n* = 37, 19.7%). Other identified causes of death included accidents (*n* = 7, 3.7%), overdose (*n* = 7, 3.7%), and murder (*n* = 3, 1.6%).

**Conclusion:**

The present analysis provides important insights into the causes of death among this hard-to-reach, marginalized group of children in LMIC. Regular surveillance of child death trends can inform targeted policy and programmatic interventions to mitigate risk and address the causes of death among young CFSW in LMICs.

## Introduction

Children of female sex workers (CFSW) in low- and middle-income countries (LMIC) are a highly vulnerable group who face numerous health and social challenges, contributing to an increased risk of mortality (1). This marginalized group of children carries an increased risk of adverse outcomes stemming from the chronic adversity and toxic stress they face growing up in the socially stigmatizing and hazardous environment of the commercial sex industry (2). They are at higher risk of exposure to food insecurity due to poverty, lack of consistent maternal income, and limited access to nutritious food (3). Malnutrition, which can lead to stunted growth, weakened immune systems, and higher susceptibility to infections, can be a major contributor to child mortality in this population (1, 4–8). A 2019 study on CFSW mortality found that nutritional deficiencies comprised the overall leading cause of death across eight LMIC, followed closely by accidents, unintentional overdoses, and communicable diseases (1).

Infectious diseases are a leading cause of under-5 mortality in developing countries, with pneumonia and respiratory illnesses as common causes, especially in communities with limited access to healthcare (1, 9–13). Additionally, diarrheal diseases are a leading driver of mortality in children globally due to a lack of access to clean water, sanitation, and health care services, seen most in marginalized populations such as CFSW (14). Furthermore, vertical HIV transmission during pregnancy, childbirth, or breastfeeding may result from high HIV prevalence among female sex workers (FSW) with limited access to prenatal care (15, 16).

While studies on specific causes of death among CFSW are scarce, studies on the causes of morbidity and mortality among FSW mothers provide clues to the exposures impacting their children. A multi-country study identified the leading causes of death among FSW in LMICs as maternal causes, followed by suicide and homicide (17). The traumatic experiences and poor health and social outcomes of FSW directly and indirectly impact the health and wellbeing of their children (18, 19). Indirectly, trauma is transmitted intergenerationally from mothers to their children, primarily through poor mental health outcomes and limited parenting and coping skills (20–24). Notably, children of FSW exhibit worse mental health functioning, report less parental monitoring, and respond differently to parenting than other children (25).

Research also highlights the direct impact of maternal trauma on the CFSW. Limited studies indicate that childhood victimization is highly prevalent among CFSW. Kidnapping, emotional, physical, and verbal abuse, neglect, sexual aggression, early separation, early sexual debut, introduction to sex work, drug use, low school enrollment, and the psychosocial impact of witnessing mothers’ interactions with clients and witnessing significant trauma such as murder were all experienced (26–28). Chronic psychological stress, violence, or trauma in childhood may impact self-regulation and relational capacities, contributing to psychiatric, emotional, and behavioral problems affecting overall health (29–33). Finally, poor sanitation and hygiene, as well as housing instability, may all contribute to mortality in CFSW (34).

This study aims to explore the causes of child mortality among CFSW in three sub-Saharan African countries: Kenya, Nigeria, and the Democratic Republic of the Congo (DRC), where previous research indicated high proportions of death among CFSW as reported by peer informants with knowledge of events in the community (1). Building on this previous work, the current study seeks to examine causes of death among CFSW by interviewing the mothers themselves about the circumstances surrounding the deaths of their children.

## Methods

### Study design and setting

In this exploratory cross-sectional study, questionnaire-based interviews with mothers who are female sex workers (MFSW) were conducted across eight cities in the sub-Saharan African countries of Kenya, Nigeria, and the DRC between February 2022 and February 2023. As part of a broader-scope parent study on maternal and child health, nutrition, mental health, and causes of mortality of urban FSW and their children, the current study leveraged established partnerships with sex worker organizations (SWO) and non-governmental organizations (NGO) to sample the hard-to-reach FSW populations in Kenya, Nigeria, and the DRC. For context, the official start and end dates of the global COVID-19 pandemic were March 2020 and May 2023, respectively, as declared by the World Health Organization (WHO).

### Study participants and recruitment

Participants were recruited by local SWO partners in three Kenyan cities (Nairobi, Mombasa, and Kisumu) and three Nigerian cities (Lagos, Calabar, and Abuja) and by NGO partners providing HIV services to FSW in the two Congolese cities of Bukavu and Kinshasa. Convenience sampling was implemented to recruit participants in locations frequented by the FSW community, such as bars, brothels, and street hotspots. Eligibility for study participation included age ≥18 years, biological mother to at least one child aged ≤5 years, engaged in sex work full-time in the past 3 years, and socially interactive with other mothers within the FSW community. Once informed about the study and determined to be eligible, participants who wished to enroll underwent an informed consent process during which voluntary participation, withdrawal from the study, and the right to decline to answer questions were reviewed. Participants indicated their consent by marking an “X” on the form. No personally identifiable information was recorded. Remuneration was in the form of two meals (before and after the study interview), childcare for participants who brought their children to the interview, and cash compensation equivalent to $16–20 USD as recommended by local partners.

### Data collection and analysis

In-person interviews were conducted in locations designated by local partners as safe and convenient for FSW. The lead investigator (BW) trained and supervised all interviewers in the eight cities. In addition to verbally administering a brief sociodemographic questionnaire, participants were asked to report on the deaths of any of their children born alive, irrespective of the time elapsed since the death. Only the data about those participants who reported the death of at least one child are included in the current analysis. Specifically, the information collected for each child death reported included the date of death, age at time of death, cause of death, circumstances surrounding the death, and HIV status, if tested. Responses were recorded on paper questionnaires in the field, then entered into an Excel database by a paid data entry consultant and reviewed for accuracy and completeness against field notes by research team members.

To facilitate quantitative descriptive statistics of the child deaths reported, deaths were organized by country, date of death, age at time of death, and cause of death. Age at time of death was categorized using WHO reporting practices: neonates (<28 days), infants (≥1 month, <1 year), under-5 (≥1 year, <5 years), and 5-to-9-year-olds. For this study, given the age of a deceased child may not have been known with exact accuracy, children <1 month were categorized as neonates or newborns. Underlying causes of death were categorized collaboratively by the lead investigator (BW) and a physician team member (WLM) using the WHO global classification (35). For example, “*the baby died due to malnutrition and inadequate breast milk*” and “*he died of starvation during the COVID outbreak*” were categorized as deaths due to ‘Nutritional deficiencies,’ whereas “*the baby died because he had yellow fever*” was coded as a death due to ‘Infectious diseases’ and “*I sent him to buy water but he got hitted [sic] by a motorcycle*” was categorized as an ‘Accident.” Death reports for which circumstantial symptoms were known without a definitive cause of death (e.g., “*he was sick for 3 days vomiting and stooling; he did not go to the hospital and died in the brothel*”) were categorized as ‘Other’ while the category of ‘Unknown’ was reserved for deaths without any details pointing to a potential cause (e.g., “*the baby was never sick; it was all so unexpected*” or “*she sleeps [sic] and never wake up*”).

## Results

### Participant characteristics

A total of 853 MFSW were recruited for the parent study, including 302 Kenyan, 301 Nigerian, and 250 Congolese MFSW. Of these 853 participants, 166 (19.5%) MFSW reported the death of at least one of their children, with a total of 203 child deaths reported. Following review of the death reports, 10 participants and 15 child deaths were excluded from the current analysis. Reasons for exclusion were as follows: five participants declined to answer questions related to the deaths of their children (i.e., no information available), two death reports had missing information about the child’s age at the time of death and/or cause of death information, one death was an abortion, one was a miscarriage, and six were stillborn deaths. Table 1 provides an overview of the participant enrollment and exclusions by study site.

**Table 1:** Study sites, interview dates, and participants included in analysis.

Country	City	Participants enrolled	Interview dates	Participants reporting child deaths	Participants excluded	Participants included in the analysis
Kenya	Nairobi Mombasa Kisumu	103 99 100	Feb 2022 Feb–Mar 2022 Mar 2022	12 (35.3%) 13 (38.2%) 9 (26.5%)	3 3 0	9 (32.15%) 10 (35.7%) 9 (32.15%)
Nigeria	Lagos Calabar Abuja	100 101 100	Mar 2022 Apr 2022 Apr 2022	19 (28.8%) 28 (42.4%) 19 (28.8%)	1 1 2	18 (29.0%) 27 (43.5%) 17 (27.4%)
DRC	Bukavu Kinshasa	100 150	Jan–Feb 2023 Feb 2023	27 (40.9%) 39 (59.1%)	0 0	27 (40.9%) 39 (59.1%)
Totals		853		166	10	156

Following the above adjustments, a total of 188 child death reports from 156 participants (28 Kenyan, 62 Nigerian, and 66 Congolese MFSW) were analyzed. The earliest reported death traced back to the year 2000. Of the 188 child deaths, 103 pre-dated the COVID-19 pandemic, while 85 (45.2%) occurred during the pandemic (between March 2020 and the last day of the study). The majority of deaths during the pandemic were reported in DRC (*n* = 51, 60.0%), and the least were reported in Kenya (*n* = 8, 9.4%).

Among the 156 MFSW who reported the death of a child, the mean age was 26.9 years (range 18–46), 24 (15.4%) were pregnant, and 26 (16.7%) reported known HIV-positive status (Table 2). The majority of MFSW (*n* = 113, 72.4%) knew their HIV status. Of the 26 HIV-positive mothers, 17 (65.4%) had their deceased children tested before their deaths, yielding 12 positive HIV tests (three in Kenya, four in Nigeria, and five in DRC). Of the 87 HIV-negative mothers, 22 (25.3%) had their children tested before death, yielding three positive tests (two Kenya, one Nigeria). Of the 43 mothers with unknown HIV status, eight (18.6%) had at least one of their deceased children tested, yielding two positive tests in DRC.

**Table 2:** Participant characteristics.

Participant characteristic	Kenya (*N* = 28)	Nigeria (*N* = 62)	DRC (*N* = 66)
Age in years, *mean*	29.8	29.1	23.5
Education level, *n (%)*			
No schooling	—	6 (9.7)	13 (19.7)
Primary	19 (67.8)	18 (29.0)	25 (37.9)
Secondary	8 (28.6)	35 (56.5)	24 (36.4)
Advanced degree	1 (3.6)	2 (3.2)	4 (6.1)
Other	—	1 (1.6)	—
Marital status, *n (%)*			
Single/never married	18 (64.3)	40 (64.5)	66 (100.0)
Separated	6 (21.4)	7 (11.3)	—
Divorced	1 (3.6)	2 (3.2)	—
Widowed	2 (7.1)	13 (21.0)	—
Declined to answer	1 (3.6)	—	—
Pregnancy status, *n (%)*			
Yes	2 (7.1)	9 (14.5)	13 (19.7)
No	26 (92.9)	53 (85.5)	53 (80.3)
HIV status, *n (%)*			
Positive	6 (21.4)	13 (21.0)	7 (10.6)
Negative	21 (75.0)	43 (69.3)	23 (34.9)
Do not know	1 (3.6)	6 (9.7)	36 (54.5)

### Child deaths by country

Table 3 offers a breakdown of child deaths by country. Of the 188 child deaths included for analysis, the largest proportion of deaths was reported in Nigeria, where 82 children were reported dead, representing a mean number of 1.32 deceased children per Nigerian mother and 43.6% of the total death reports. The DRC followed with 74 child deaths (39.4%), and Kenya with 32 deaths (17.0%). The overall mean number of deceased children per mother included in the study was 1.21, with most participants (*n* = 131, 84.0%) reporting the death of a single child, 19 (12.2%) MFSW each reporting two child deaths, 5 (3.2%) reporting three child deaths, and 1 (0.6%) Nigerian mother reporting the deaths of four of her children, ages 2–6 years.

**Table 3:** Child deaths by country.

Country	No. MFSW participants (% total)	No. child deaths reported	% of child deaths reported	Mean child deaths per participant (range)	Age range of deceased children
Kenya	28 (18.0)	32	17.0%	1.14 (1–3)	<1 day to 9 years
Nigeria	62 (39.7)	82	43.6%	1.32 (1–4)	<1 day to 7 years
DRC	66 (42.3)	74	39.4%	1.12 (1–2)	<1 day to 8 yrs. 2 mo
Total	156	188	100%	1.21	

### Child deaths by country and age group

Neonates or newborns (age <1 month) and infants (age ≥1 month, <1 year) together were overrepresented in the pooled death counts across countries (*n* = 134, 71.3%). In Kenya and Nigeria, the preponderance of reported deaths involved neonates (46.9 and 41.4%, respectively), while infants accounted for the largest proportion of deaths in the DRC (*n* = 36, 48.7%). The largest proportions of deaths among neonates, under-5, and 5- to 9-year olds were reported in Nigeria (43.9%). Of the 69 newborn deaths, over three-quarters died within the first week of life (*n* = 53, 76.8%) and nearly half (*n* = 34, 49.3%) lived no longer than 24 h after birth. Tables 4, 5 provide further detailed information on deaths by country and age group.

**Table 4:** Child deaths by country and age group.

Child age group	Kenya		Nigeria		DRC		Total *N* (*% all deaths*)
	$n\;\frac{\%\text{deaths}\;\;\text{in}\;\;\mathrm{age}\;\;\text{group}}{\%\text{deaths}\;\;\text{in}\;\;\text{country}}$						
Neonate (birth to <1 mo)	15	21.7	34	49.3	20	29.0	69 (36.7)
		46.9		41.4		27.0	
Infant (≥ 1mo to <12 mo)	8	12.3	21	32.3	36	55.4	65 (34.6)
		25.0		25.6		48.7	
Under-5 (≥12 mo to <5 yr)	7	17.1	18	43.9	16	39.0	41 (21.8)
		21.9		22.0		21.6	
5–9 years (≥5 yr to <10 yr)	2	15.4	9	69.2	2	15.4	13 (6.9)
		6.2		11.0		2.7	
Total *N* (*% all deaths in country*)	32 (17.0)		82 (43.6)		74 (39.4)		188

**Table 5:** Neonatal deaths by country and newborn group.

Neonatal age group	Kenya		Nigeria		DRC		Total deaths (% *all neonatal deaths*)
	$n\;\frac{\%\text{deaths}\;\mathrm{age}\;\text{group}}{\%\text{deaths}\leq 7-\mathrm{day}\;\text{in country}}$						
Newborn ≤ 1 day	6	17.6	20	58.8	8	23.5	34 (49.3)
		60.0		71.4		53.3	
Newborn ≥ 2 days, ≤ 7 days	4	21.1	8	42.1	7	36.8	19 (27.5)
		40.0		28.6		46.7	
Newborn > 7 days	5	31.25	6	37.5	5	31.25	16 (23.2)
		0		0		0	
Total newborn deaths *N* (*% all child deaths in country*)	15 (46.9)		34 (41.5)		20 (27.0)		69

### Child deaths by cause, country, and age group

Table 6 details reported deaths by cause of death, country, and age group. The overall leading cause of death reported across the three study countries was neonatal conditions (*n* = 50, 26.6%), which comprised 72.5% of all neonatal deaths and included conditions such as premature birth, labor and delivery complications (e.g., prolonged labor, asphyxia, aspiration of amniotic fluid), neonatal infection, and failure to feed and thrive. Infectious diseases (*n* = 41, 21.8%) comprised the second overall leading cause of death in CFSW. They included infections such as malaria (*n* = 13, 31.7%) and yellow fever (*n* = 2, 4.9%) as well as diseases with person-to-person transmission such as measles (*n* = 10, 24.4%), HIV (*n* = 7, 17.1%), pneumonia (*n* = 5, 12.2%), tuberculosis (TB; *n* = 2, 4.9%), typhoid fever (*n* = 1, 2.4%), and “a virus” (*n* = 1, 2.4%). Deaths due to infectious diseases were disproportionately reported among infants (*n* = 20, 30.8%) and children under age 5 (*n* = 14, 34.1%), accounting for the single most reported cause of child death in each of these age groups. Table 7 provides a breakdown of infectious diseases by country and age group.

**Table 6:** Causes of child death by country and child age group.

Cause of death	Kenya					Nigeria					DRC					Total (*by cause & age*)				
	N	I	U5	≥5	T	N	I	U5	≥5	T	N	I	U5	≥5	T	N	I	U5	≥5	T (%)
Accidents			2		2		1	1		2		2		1	3	0	3	3	1	7 (3.7)
Infectious diseases	1	5	4	2	12	1	6	6	2	15	1	9	4		14	3	20	14	4	41 (21.8)
Murder					0					0	2		1		3	2	0	1	0	3 (1.6)
Neonatal conditions	14				14	26				26	10				10	50	0	0	0	50 (26.6)
Nutritional deficiencies					0	1	4	7	4	16	3	12	5	1	21	4	16	12	5	37 (19.7)
Other		1			1	2	5	2	2	11	2	6	3		11	4	12	5	2	23 (12.2)
Overdose					0	1	1			2	2	2	1		5	3	3	1	0	7 (3.7)
Unknown		2	1		3	3	4	2	1	10		5	2		7	3	11	5	1	20 (10.6)
Total *(by country & age)*	15	8	7	2	32	34	21	18	9	82	20	36	16	2	74	69	65	41	13	188

N, newborn; I, infant; U5, under 5 years old; ≥5, 5 years and older; T, total. The highlights indicate the leading cause of death by country, irrespective of age group.

**Table 7:** Deaths due to infectious diseases by country and child age group.

Infectious disease	Kenya					Nigeria					DRC					Total (*by cause & age*)				
	N	I	U5	≥5	T	N	I	U5	≥5	T	N	I	U5	≥5	T	N	I	U5	≥5	T (%)
HIV					1		1	2		3		3			3	0	4	3	0	7 (17.1)
Malaria		4	1	2	7		1	2		3	1	1	1		3	1	6	4	2	13 (31.7)
Measles			1		1		1	2	2	5		3	1		4	0	4	4	2	10 (24.4)
Pneumonia	1	1			2	1	2			3					0	2	3	0	0	5 (12.2)
Tuberculosis			1		1					0			1		1	0	0	2	0	2 (4.9)
Typhoid					0		1			1					0	0	1	0	0	1 (2.4)
Virus					0					0		1			1	0	1	0	0	1 (2.4)
Yellow fever					0					0		1	1		2	0	1	1	0	2 (4.9)
Total (*by country & age*)	1	5	4	2	12	1	6	6	2	15	1	9	4	0	14	3	20	14	4	41

N, newborn; I, infant; U5, under 5 years old; ≥5, 5 years and older; T, total. The highlights indicate the leading cause of death by country, irrespective of age group.

Nutritional deficiencies, reported by participants as “malnutrition,” comprised the third overall leading cause of death (*n* = 37, 19.7%) across the study. However, they were not reported at all as a cause of death in Kenya. While nutritional deficiencies were responsible for the largest proportion of all child deaths in DRC (*n* = 21, 28.4%), neonatal conditions were the leading cause of death in Kenya (*n* = 14, 43.8%) and Nigeria (*n* = 26, 31.7%) but comprised a smaller proportion (*n* = 10, 13.5%) of all child deaths in DRC. The next most common overall causes of death were coded as “Other” (*n* = 23, 12.2%) and “Unknown” (*n* = 20, 10.6%). Although contextual details were known about deaths in the “Other” category (e.g., anemia, convulsions, death while sleeping, dehydration, fevers, gastrointestinal bleeding, vomiting, and diarrhea), the available information was not sufficient to determine the cause of death with any certainty. In the “Unknown” death category, no clinical symptoms or other details of the death were reported.

Aside from the top five overall leading causes of death described, a smaller proportion of the overall deaths reported were due to accidents (*n* = 7, 3.7%; three house fires, one pedestrian struck by a motor vehicle, one fall out of bed, one collateral harm due to violence), overdoses (*n* = 7, 3.7%; unintentional oversedation by mother needing to work), and murder (*n* = 3, 1.6%; acid burn, poisoning, abusive trauma).

## Discussion

This cross-sectional study offers important insights into causes of child death among a hard-to-reach, marginalized group of children in Kenya, Nigeria, and the DRC for whom scant mortality data are available (1). Our analysis found that nearly one in five MFSW had experienced the loss of a young child—a stark statistic highlighting the need for research to understand and address preventable causes of mortality in CFSW. The current study facilitates a greater understanding about CFSW mortality by interviewing the mothers themselves about the circumstances surrounding the deaths of their children, such as presenting symptoms or contributing factors. Furthermore, given the timing of the investigation, this study may capture causes of death attributable to the COVID-19 pandemic. Short of conducting formal verbal autopsies, such real-time analyses of maternal reports on the causes of child death can be especially useful for governments and organizations looking to inform timely responses and implement appropriate and effective interventions as concerning trends arise.

Deaths of children under 1 year of age accounted for 71.3% of all child deaths in the current study, with newborn deaths (<1 month old) outnumbering infant deaths (36.7% versus 34.6%, respectively), and neonatal conditions (72.5%) representing the primary cause of newborn deaths. At a country level, neonatal conditions represent the single leading cause of death among all under-1-year deaths in Kenya (60.9%) and Nigeria (47.3%) in this study, while nutritional deficiencies accounted for the single leading cause of under-1-year deaths (21.4%) in the DRC, followed by neonatal conditions (17.9%). The high level of mortality due to neonatal conditions and the detailed death accounts provided by mothers appear to support previous research documenting barriers to maternal care and poor pregnancy outcomes among MFSW (36–39).

Consistent with study findings, neonatal conditions (preterm birth complications, perinatal asphyxia and trauma, and neonatal sepsis and infections) accounted for the largest proportion of under-1-year deaths per 100,000 in the general population of each of the three study countries, according to 2021 WHO global estimates on leading causes of death (40). As reflected by the annual 2.5 million neonatal deaths documented in LMIC globally, the immediate postnatal period through the first month of life constitutes a critical period that carries a significantly high risk for mortality (41). Although information about antenatal care (ANC), birth location, and the presence of skilled birth attendants (SBA) was not systematically collected, the disproportionate neonatal death count among CFSW may be attributable to the documented decreased access to ANC and SBA among FSW (42–47). In general, maternal health care services are challenging for MFSW to access and afford in LMIC, and it is possible that the COVID-19 pandemic exacerbated these barriers through loss of income, delays in accessing care, facility lockdowns, and attrition of providers, including SBA (48–50).

In contrast to the current study, neonatal conditions comprised a smaller proportion of under-1-year deaths in the 2019 study on causes of CFSW death (1), where the leading causes of death were accidents in Kenya (34.0%) and overdoses in Nigeria (55.6%) and DRC (58.6%). This earlier study described fatal accidents and overdoses as occurring in the setting of MFSW, leaving their infants unattended at home or administering substances to induce sleep in their children, practices that make it possible for new mothers to earn income by returning to sex work (1). With nearly half the deaths documented in this study occurring after the onset of the pandemic, it is possible that fatal accidents and overdoses in the under-1 age group were less frequent in the current study due to diminished work opportunities during the pandemic.

The risk of fatal accidents early in life may be uniquely elevated in high-risk populations such as CFSW. In the current study, 4.6% of infant deaths were due to house fires, falls from height, and collateral injury from violence directed at others. Nevertheless, this percentage of deaths is drastically lower than that due to accidents reported in the 2019 study (1). Unlike CFSW, however, accidents are not found among the WHO top 10 leading causes of infant death in any of the three study countries (40). These findings corroborate previous research documenting the adversity these children face starting at a young age (26–28).

Among infants (≥1 month and <1 year old), infectious diseases were overall responsible for the largest proportion of deaths by a single cause (*n* = 20, 30.8%), followed by nutritional deficiencies (*n* = 16, 24.6%). In DRC, however, deaths due to nutritional deficiencies outnumbered those due to infectious disease by 25% (*n* = 12 versus *n* = 9, respectively). In comparison to findings from the 2019 CFSW study (1), infectious diseases, at their highest placement, ranked third among leading causes of infant death in DRC only.

Similarly, among children under 5 years, infectious diseases were responsible for the largest proportion of overall deaths by a single cause (*n* = 14, 34.1%). Disaggregated by country, infectious diseases comprised the leading cause of under-5 death in Kenya only. In Nigeria and the DRC, deaths due to nutritional deficiencies slightly outnumbered infectious disease deaths in this age group. For comparison, in the 2019 CFSW mortality study, infectious diseases were responsible for the largest proportion of under-5 deaths in Nigeria only. They ranked second to accidents in Kenya and nutritional deficiencies in the DRC (1). While the combined infant and under-5 findings in the current study suggest that infectious diseases may have gained prominence as a preventable cause of death among CFSW since the 2019 study, the findings confirm that deaths due to nutritional deficiencies remain an important cause of death to address.

Infectious diseases identified in this study included HIV, malaria, measles, TB, and yellow fever. Interestingly, although measles was reported as a cause of death in this age group by mothers from all three countries (4 out of 14, 26.6%), global estimates place measles among the top 10 leading causes of under-5 death in the DRC only (40). While additional research is needed to determine the significance of this difference, the current data suggest that Kenyan and Nigerian under-5 CFSW may be different from their counterparts in the general population. Compared to the general child population, CSFW may have lower vaccination rates due to limited access to health care (51–55) and higher levels of pathogen exposures and transmission due to more hazardous, crowded living conditions (28, 56, 57). Similarly, HIV was reported as a cause of infant death in DRC and Nigeria, whereas global estimates only place HIV in the top 10 leading causes of under-1-year deaths in Kenya (40). This discrepancy raises the possibility that at least one of the following may be true for this high-risk population as compared to the general population: pregnant FSW have less access to HIV vertical-transmission prevention, CFSW are more likely, if tested, to be tested for HIV in infancy, CFSW have a higher incidence of HIV, and CFSW have decreased access to HIV treatment. Further research is needed to verify the validity of these findings and identify potential factors contributing to any discrepancies detected. Finally, although the etiology of deaths categorized as ‘Other’ remains unclear, these deaths comprised the fourth leading cause of CFSW death and the associated symptoms described by mothers could be associated with infectious diseases—e.g., convulsions (meningitis), fevers (malaria), bleeding manifestations (yellow fever), and vomiting/diarrhea (typhoid)—and merit further investigation.

Although nutritional deficiencies comprised the second leading cause of death among under-5 deaths in the current study, they are only included in the WHO top 10 leading causes of under-5 death in Kenya (40)—again, suggestive of a potential significant difference in the leading causes of death among CFSW and other children. In this case, young CFSW may experience higher and more severe levels of food insecurity at baseline—even without the added strain of a global pandemic—as compared to the general child population (3, 58–60). Among the 13 reported deaths of older 5- to 9-year-olds in this study, nutritional deficiencies (38.5%) comprised the majority of child deaths. Whereas nutritional deficiencies are a less prominent cause of death among children five and older according to WHO estimates for Kenya and DRC, nutritional deficiency deaths are not in the WHO top 10 causes of death among 5- to 9-year-olds in Nigeria. Furthermore, according to the WHO data, children aged five and older have a lower risk of death than children under 5 years of age (58). The much lower proportion of deaths among children 5 years and older (6.9%) in this cohort is consistent with the steep decline observed in global death rates among older children 5–9 years old as compared to the under five group (61).

While murder was reported as a cause of death among Congolese newborns and children under 5 in this cohort, interpersonal violence does not make the top 10 leading causes of death list until the 10- to 14-year-old age group in Kenya and the 15- to 19-year-olds in DRC and Nigeria (40). These discrepancies are consistent with the 2019 study on CFSW deaths (1) and suggest an underlying difference between the risks and exposures of CFSW ages versus the general child population starting at a very young age. The extant literature corroborates a generally heightened level of danger and violence in the living conditions of CFSW as compared to other less marginalized child populations (26, 62).

Finally, preliminary time-phased analyses suggest that the cause-of-death distribution in Nigeria may be statistically different in the pandemic years (March 2020 and beyond) as compared to the pre-pandemic years (*p = 0.03*, analyses not shown). However, it is important to note that the disaggregation of deaths by country, cause, and time phase resulted in cause-of-death sample sizes too small for sound statistical comparison. Therefore, further research with a larger random sample of child deaths is needed to reliably determine the presence of such trends.

### Strengths and limitations

The main strength of this study is the use of questionnaire-based interviews with the individuals who would be most knowledgeable about the circumstances and causes of death among children in the community—the mothers themselves. As a result, recall bias may be lessened by the fact that mothers are less likely to forget the death of their own children. However, memory of the details surrounding these deaths may wane or change with time, and the potential for the misclassification of stillbirths as early neonatal deaths cannot be excluded. Moreover, despite employing a conservative coding approach in which ambiguous causes of death were categorized as ‘Other,’ the level of confidence around the maternal assertion of cause of death must be considered. Although research suggests that mothers are reliable informants of causes of death in the community (1, 63–66), efforts to triangulate the accuracy of the cause of death could not be undertaken.

It is important to note that due to convenience sampling, the findings of this study may not be representative of all CFSW in these countries. While measures to reduce sampling bias may be employed, it is nearly impossible to fully eliminate this type of bias when studying marginalized or criminalized hard-to-reach populations. As previously noted, additional research employing random sampling techniques is needed to determine the significance of any differences in causes of death between CFSW and the general child population in any given country.

Lastly, selection bias cannot be excluded as there may be elemental differences between MFSW who choose to participate and those who decline study participation. Similarly, the use of only mothers as informants of child death introduces bias by systematically excluding information on the deaths of children whose mothers are deceased. Furthermore, death counts and details of the reported deaths may be impacted by social desirability bias, as participants may seek to gain favor or avoid judgment, particularly when communicating more sensitive information, such as details of the deaths of their children.

## Conclusion

Children reared by mothers who are female sex workers grow up in the same high-risk, marginalized environments in which their mothers live and work. While the findings of this study parallel WHO 2021 country data on neonatal conditions and infectious diseases as leading causes of child death, deaths due to nutritional deficiencies, accidents, overdoses, and murder hold more prominence and suggest that CFSW carry a higher risk of adverse exposures and outcomes at earlier ages. Deaths due to nutritional deficiencies raise concern for severe food insecurity, disproportionate to their counterparts in the general population, and perhaps exacerbated in the peri-pandemic years among CFSW. Additionally, the role of neglect, exposure to violence and abuse, unsafe brothel- or street-based living, and inadequate access to newborn care and preventative health care (e.g., childhood vaccinations) in CFSW mortality merits future investigation to guide prevention strategies and inform effective interventions. Coordinating large-scale mortality surveillance efforts within the hard-to-reach CFSW populations in these countries is key to identifying and tracking unique trends in cause of death. Such real-time data can assist government ministries, intergovernmental bodies, non-governmental organizations, and funders in the timely development of policies and allocation of resources for evidence-based programs that reduce preventable deaths, improve health and wellbeing, and extend the lifespan of these at-risk children.

## Data Availability

Deidentified aggregate data used for this analysis is presented in the article. Additional inquiries can be directed to bwillis@globalhealthpromise.org, who will consider requests for the purpose of a research partnership or provision of services to female sex workers and their children.
